# Sustained treatment-free remission in two chronic myeloid leukemia patients after asciminib discontinuation: a report of two cases

**DOI:** 10.1007/s00277-024-06170-4

**Published:** 2025-01-10

**Authors:** Arjen Yousefi, Mark-David Levin, Jan J. Cornelissen, Peter E. Westerweel

**Affiliations:** 1 Department of internal medicine, Albert Schweitzer Hospital, Dordrecht, The Netherlands; 2 Department of hematology, Erasmus Medical Center, Rotterdam, The Netherlands

**Keywords:** Chronic myeloid leukemia, Treatment-free remission, Asciminib, TKI withdrawal syndrome

## Abstract

Selected chronic myeloid leukemia (CML) patients may discontinue their tyrosine kinase inihibitor (TKI) in an attempt to achieve sustained treatment-free remission (TFR), which mitigates therapy-related side effects and limits treatment costs. TFR has been extensively studied following the discontinuation of adenosine triphosphate (ATP) – competitive TKI. However, there is minimal data concerning TFR after the discontinuation of the novel TKI asciminib. Here, we present two CML patients intolerant to multiple ATP-competitive TKIs who achieved a deep molecular response (DMR) during asciminib treatment and sustained this remission after asciminib discontinuation. One of the cases developed transient myalgia and arthralgia after asciminib discontinuation consistent with a TKI withdrawal syndrome. Both patients have been free of molecular relapse for more than at least 8 months after TKI discontinuation without increase in molecular BCR::ABL1 signal. These two cases provide proof of principle that sustained TFR after discontinuing asciminib in CML patients is feasible.

## Introduction

Treatment-free remission (TFR) has become an important goal to alleviate therapy-related side effects and limit costs in chronic myeloid leukemia (CML). The 2020 European LeukemiaNET (ELN) guidelines recommend that selected chronic phase CML patients in stable deep molecular response (DMR) may consider discontinuing their tyrosine kinase inhibitor (TKI) therapy under frequent monitoring for molecular relapse [1]. Eligibility criteria include (a) first-line therapy or second-line if intolerance was the only reason for changing TKI; (b) typical e13a2 or e14a2 BCR::ABL1 transcripts; (c) duration of TKI therapy > 5 years (> 4 years for second generation TKI); (d) duration of DMR (MR4 or better: a BCR::ABL1 ≤ 0.01% on the international scale (IS)) > 2 years and (e) no prior treatment failure. Using these selection criteria, approximately half of the stopped patients will sustain the TFR, while the other half will experience a molecular relapse at a BCR::ABL1 > 0.1% on the IS, requiring the resumption of TKI therapy. If a molecular relapse occurs, this mostly takes place during the first 6 months after TKI discontinuation [2].

It is important to note that all studies that formed the basis of these recommendations involved discontinuation of adenosine triphosphate (ATP) – competitive TKIs, namely imatinib, dasatinib, nilotinib and bosutinib. Currently, there is minimal data on CML patients attempting TFR after discontinuation of the novel TKI asciminib. Asciminib uniquely targets the myristoyl pocket of the BCR-ABL1 oncoprotein, in contrast to ATP-competitive TKIs, which target the kinase domain [3]. This distinct mechanism of action provides superior molecular response rates and tolerability, resulting in FDA and EMA registration for treatment of CML patients with resistance or intolerance for at least two ATP-competitive TKIs [4]. It is unclear if CML patients treated with asciminib may also be eligible to attempt TFR using similar selection criteria as have been developed for ATP-competitive TKI. Here, we describe the first two cases of CML patients with BCR::ABL1 transcripts quantifiable on the IS achieving a sustained TFR after discontinuing asciminib.

## Case 1

This is a 75 year old male who was diagnosed with CML, presenting with a leukocytosis of 158 × 10^9^ /L, 3% basophils, 1% blasts, 571 × 10^9^ /L platelets and a splenomegaly of 1 cm below the arcus costarum. The bone marrow examination was consistent with chronic phase CML with cytogenetic analysis confirming the presence of the Philadelphia chromosome. No high-risk additional chromosomal abnormalities were found. Multiplex PCR detected an e13a2 BCR::ABL1 transcript type. He was at intermediate risk according to the EUTOS long-term survival (ELTS) score and was initially treated with imatinib 400 mg QD for a period of 6 months. However, he discontinued it because of Common Terminology Criteria for Adverse Events (CTCAE) version 5.0 grade 2 myalgia and tremor [5]. The patient was then switched to nilotinib 300 mg BID which he had to discontinue after 9 months of treatment due to grade 2 coughing and dyspnea. His final ATP-competitive TKI was dasatinib, dosed at 100 mg QD, which he discontinued after 1 month of treatment due to again grade 2 coughing and dyspnea. With a total treatment duration of 1 year and 4 months and intolerance for three ATP-competitive TKIs, the patient was started on asciminib 40 mg BID. The BCR::ABL1 was 0.011% IS at the initiation of asciminib treatment. The patient achieved a MR4.5 with a BCR::ABL < 0.003% after 3 months of treatment and undetectable BCR::ABL1 on Cepheid Xpert BCR-ABL Ultra molecular platform after 15 months of treatment. He continued to receive asciminib for a total duration of 2 years with good tolerability and absence of adverse events. After 2 years of asciminib treatment and total TKI treatment period of 3.5 years, the decision was made to attempt to discontinue asciminib. The depth of remission prior to asciminib discontinuation was confirmed with digital droplet PCR, which showed a BCR::ABL1 of 0,0006% on the IS [6]. Two weeks after asciminib was discontinued, the patient developed a TKI withdrawal syndrome with generalized myalgia and arthralgia. Paracetamol and non-steroidal anti-inflammatory drugs were insufficient to alleviate the symptoms, but these responded well to oral corticosteroids. At the time of last follow-up, 8 months after asciminib discontinuation, the BCR::ABL1 remained undetectable by real-time quantitative PCR (RT-qPCR) using the Cepheid Xpert BCR-ABL Ultra test with a limit of detection at 0.0030% on the IS.

## Case 2

This is a 68 year old female who was diagnosed with CML with a leukocytosis of 311 × 10^9^ /L with 3% basophils, 9% blasts, 486 × 10^9^ /L platelets and a splenomegaly of 5 cm below the arcus costarum. The bone marrow examination was consistent with chronic phase CML. Multiplex PCR detected an e14a2 BCR::ABL1 transcript type. She was classified as high-risk based on the ELTS score and she was treated with imatinib 400 mg QD with an initial optimal hematological and molecular response. However, she experienced CTCAE grade 2 edema and grade 1 hyperpigmentation and therefore discontinued imatinib after 9 months of treatment. She then switched to nilotinib 300 mg BID as second line of treatment. This was taken for a period of 7 months with an initial optimal molecular response, but again discontinued due to intolerance with grade 2 pruritus, grade 2 skin rash, grade 2 anemia, and grade 3 thrombocytopenia. With an intolerance of two ATP-competitive TKIs, she was enrolled into the open label phase III-ASCEMBL trial and was randomized to receive asciminib 40 mg BID [4]. The asciminib was well tolerated at the standard dose with only grade 1 skin en nail toxicity. Baseline BCR::ABL1 after a period of nilotinib discontinuation had been 14% IS, but she achieved a major molecular remission after 9 months of asciminib treatment and an MR4.0 after 3 years. After a total asciminib treatment duration of 5 years and 9 months and total TKI treatment duration of 7.5 years, the decision was made to discontinue asciminib. BCR::ABL1 was < 0.003% on RT-qPCR. The depth of remission was confirmed by digital droplet PCR at 0.0050% IS. After asciminib discontinuation, the patient did not experience TKI withdrawal symptoms. At the time of last follow-up, 8 months after asciminib discontinuation, the BCR::ABL1 remained detectable, but not quantifiable as it remained below the limit of detection of 0.0030% on the IS using the Cepheid Xpert BCR-ABL Ultra test.

## Discussion

Our case reports are the first to describe two chronic phase CML patients with typical e13a2 and e14a2 BCR::ABL1 transcripts who maintained a stable TFR for at least 8 months after discontinuing asciminib. With this, we provide a proof of principle that sustained TFR is feasible after asciminib treatment. Interestingly, we are the first to report occurrence of a TKI withdrawal syndrome after asciminib discontinuation.

To date, few cases of attempted TFR after asciminib discontinuation have been reported. Yoshimaru et al. [7] described four CML patients who all experienced a molecular relapse in the first 4 months after discontinuing asciminib. Of note, all four patients had a history of resistance to ATP-competitive TKI. Ernst et al. [8] reported a patient who remained free of molecular relapse after asciminib discontinuation despite earlier development of a T315I kinase domain mutation and ponatinib resistance. In addition, the patient had an atypical BCR::ABL1 transcript. Given the previous TKI resistance and the atypical transcript type, the patients reported by Yoshimaru et al. [7] and Ernst et al. [8] would not qualify for a TFR attempt according to the ELN guideline [1]. Previous resistance to TKIs is associated with lower sustained TFR-rates according to two second-line TKI discontinuation trials [9, 10].

The two patients reported by us may be considered more typical candidates for a TFR attempt, based on the eligibility criteria in the ELN guideline [1]. Our cases have had a follow-up of at least 8 months after asciminib discontinuation. This is of importance, as the probability of a molecular relapse is the highest in the first 6 months after a TKI discontinuation [2]. However, further follow-up is needed to establish long-term durability of TFR after asciminib treatment and discontinuation.

One of our two cases developed a transient syndrome of myalgia and arthralgia after asciminib discontinuation. This is consistent with the clinical TKI withdrawal syndrome previously reported to occur in approximately 20–30% of patients discontinuing an ATP-competitive TKI [1, 11]. In the Korean Imatinib Discontinuation Study, the occurrence of TKI withdrawal syndrome was associated with a sustained TFR without molecular relapse [12]. The withdrawal syndrome has been speculated to be caused by off-target effects of TKI, but it may also result from an immunological response. As asciminib has significantly fewer off-target effects than ATP-competitive TKI, it is surprising to note a TKI withdrawal syndrome after asciminib discontinuation if this would be the mechanism.

In conclusion, CML patients in sustained DMR, with a history of TKI intolerance but no prior TKI resistance, may achieve sustained TFR following asciminib discontinuation. Further clinical trials are required to better establish the safety, proportion of patients with sustained TFR, kinetics of molecular relapse and optimal eligibility criteria for TKI discontinuation of asciminib compared with current ATP-competitive TKI.

## Data Availability

No datasets were generated or analysed during the current study.
